# Virtual Signal Processing-Based Integrated Multi-User Detection

**DOI:** 10.3390/s25154761

**Published:** 2025-08-01

**Authors:** Dabao Wang, Zhao Li

**Affiliations:** 1 Institute of Remote Sensing Satellite, China Academy of Space Technology, Beijing 100094, China; cast_wdb@126.com; 2 School of Cyber Engineering, Xidian University, Xi’an 710126, China; 3 School of Electronics and Information, Zhengzhou University of Light Industry, Zhengzhou 450001, China

**Keywords:** multi-user detection (MUD), signal processing, bit-error rate (BER)

## Abstract

The demand for high data rates and large system capacity has posed significant challenges for medium access control (MAC) methods. Successive interference cancellation (SIC) is a classical multi-user detection (MUD) method; however, it suffers from an error propagation problem. To address this deficiency, we propose a method called *Virtual Signal Processing-Based Integrated Multi-User Detection (VSP-IMUD)*. In VSP-IMUD, the received mixed multi-user signals are treated as an equivalent signal. The channel ambiguity corresponding to each user’s signal is then examined. For channels with non-zero ambiguity values, the signal components are detected using zero-forcing (ZF) reception. Next, the detected ambiguous signal components are reconstructed and subtracted from the received mixed signal using SIC. Once all the ambiguous signals are detected, the remaining signal components with zero ambiguity values are equated to a virtual integrated signal, to which a matched filter (MF) is applied. Finally, by selecting the signal with the highest channel gain and adopting its data as the reference symbol, the remaining signals’ dataset can be determined. Our theoretical analysis and simulation results demonstrate that VSP-IMUD effectively reduces the frequency of SIC applications and mitigates its error propagation effects, thereby improving the system’s bit-error rate (BER) performance.

## 1. Introduction

The ever-increasing demand for wireless broadband services has driven the development of 5G technology. Compared to previous mobile communication systems, 5G is expected to offer larger system capacity, higher data rates, lower latency, and improved reliability [1]. As a key application in the 5G era, the Internet of Things (IoT) has been rapidly developing in recent years, leading to explosive growth in the number of IoT devices. It is estimated that by 2025, more than 41.6 billion IoT devices will be connected to networks worldwide [2,3]. Therefore, efficiently supporting the data transmissions of more users under limited communication resources has become a critical issue warranting thorough investigation.

By allocating different types of communication resources—such as frequency, time slots, code words, and spatial characteristics—to multiple users in an orthogonal manner, interference among concurrent data transmissions can be effectively avoided, thereby enabling resource sharing among users [4]. However, since the frequency resources available for wireless communication are limited, the emergence of various communication systems and the increase in mobile users pose significant challenges to traditional orthogonal multiple access (OMA) methods. As a result, dynamic spectrum sharing methods have been proposed to facilitate multi-user communications. For example, cognitive radio (CR) [5,6] enables unlicensed or cognitive users to access the available authorized spectrum for data transmission. However, timely sensing of the licensed user’s signal and withdrawal is critical for CR applications, as inappropriate spectrum access may interfere with authorized communications. Due to advancements in microelectronic technology, the processing capability of hardware has improved while costs have decreased. A receiver (Rx) can now decode data carried in multiple concurrent signals through complex signal processing. In recent years, non-orthogonal multiple access (NOMA) [7] has emerged as a promising medium access control (MAC) method for the 5G system. Uplink NOMA allows multiple transmitters (Txs) to transmit to a common Rx over the same frequency channel in a non-orthogonal manner, enabling the Rx to employ successive interference cancellation (SIC) [8] to mitigate co-channel interference (CCI).

As mentioned above, in addition to resource sharing, receiver-side multi-user detection (MUD) is essential for achieving high system capacity and data rates. Existing MUD methods can be classified into linear detection and non-linear detection based on whether their structures involve feedback mechanisms. Linear MUD methods include linear de-correlation detection [7] and minimum mean squared error (MMSE) detection [8]. However, linear MUD [9] requires computing the inverse of the cross-correlation matrix of all users’ channels, which incurs high computational complexity. Non-linear MUD, on the other hand, includes decision feedback detection methods such as SIC. SIC first recovers data information carried in certain signal components from the received mixed signal, then uses the decoded data and estimated channel state information (CSI) to reconstruct the previously detected signals. These reconstructed signals are subtracted from the received mixed signal, resulting in a reduced mixed signal. By performing this iterative processing on the reduced mixed signal, multiple users’ signals can be detected. By combining MMSE and SIC, MMSE-SIC detection has been proposed and applied within the V-BLAST framework [10,11]. However, SIC has an error propagation problem [12], leading to a high bit-error rate (BER) for later-decoded users’ data, which hampers its practical application.

Although increasing the number of receiving antennas can enhance the Rx’s signal processing capability and support more users’ data transmissions, practical limitations such as device size and hardware costs make it unfeasible to increase the number of receiving antennas indefinitely. Therefore, utilizing a limited number of receiving antennas for multi-user detection (MUD) holds significant research importance. By exploiting interactions among multiple wireless signals, the authors of [12] proposed a MUD scheme named ICom/SIC-ZF, which incorporates interference combination (ICom) with zero-forcing (ZF) reception and SIC. This method treats all signals other than the desired signals (i.e., those to be detected) as a structured equivalent interference, and uses ZF to cancel this interference while recovering the desired signals. Subsequently, SIC is employed to subtract the detected signals from the received mixed signal. By iterating this process on the remaining mixed signal, all users’ signal components can be detected sequentially. In [13], the authors utilized maximum likelihood (ML) to decode data from two overlapping wireless signals simultaneously under a single receiving antenna setting, based on the characteristics of various combinations of symbols carried in the mixed signal. However, with high-order modulation or a large number of users’ signals, multiple symbol combinations can produce identical constellation points, leading to decoding errors. In such cases, the BER performance of this method diminishes significantly. The authors of [14] utilized intelligent reflecting surfaces (IRSs) to reflect a transmitted signal towards the intended Rx. By intelligently adjusting the reflecting parameters of the IRS, the reflected signal can interact with the directly arriving component to produce a desired signal at the Rx. Since neither the directly transmitted nor the reflected signal component carries legitimate data information, it becomes difficult or even impossible for an eavesdropper to intercept the legitimate user’s information. In [15], an IRS was employed to reflect interference towards the affected Rx. The reflected interference serves as a recycling signal that interacts with the direct interference to generate a desired signal, thereby achieving interference recycling and enhancing data transmission.

Based on the above discussions, this paper proposes a method called *Virtual Signal Processing-Based Integrated Multi-User Detection (VSP-IMUD)*. This approach achieves *integrated MUD (IMUD)* by leveraging the spatial features of the equivalent desired signals that consist of multiple users’ signals. First, we design a *channel ambiguity detection (CAD)* module to avoid similarity or ambiguity among different equivalent (virtual) desired signal structures. In scenarios where similarity or ambiguity exists, directly applying IMUD may result in decoding errors and poor BER [13]. The CAD module identifies channels that yield ambiguity and then employs ICom/SIC-ZF to detect signals transmitted through these ambiguous channels. Once all signals transmitted through the ambiguous channels are decoded, the remaining signal components are treated as an equivalent desired signal through virtual signal combination. Based on the spatial characteristics of this equivalent desired signal, we can derive possible symbol combinations of the various desired signal components. Finally, we use ZF to recover the data sent from the Tx with the largest channel gain relative to the common Rx, and adopt it as the reference data to determine the exact symbol set of the equivalent signal. In this way, VSP-IMUD is realized.

The contributions of this paper are two-fold:

- Proposal of VSP-IMUD. This method allows for the integral decoding of data information carried in multiple signal components at once, based on the overall spatial characteristics of the received mixed signal. This enables the common Rx to perform multi-user detection (MUD) with fewer receiving antennas.
- Proposal of CAD. This module first identifies channels that cause the structure of one equivalent desired signal to be ambiguous relative to another. By detecting signals transmitted through these ambiguous channels, the uniqueness of the remaining signals’ equivalent structure is ensured, allowing for IMUD to effectively decode multiple users’ data simultaneously.

The rest of this paper is organized as follows. Section 2 describes the system model, while Section 3 details the design of VSP-IMUD. Then, Section 4 evaluates the performance of the proposed scheme. Finally, Section 5 concludes the paper.

Throughout this paper, we will use the following notations. Let C denote the set of complex numbers. Vectors and matrices are represented by bold letters. $X^{T}$, $X^{H}$ and $X^{-1}$ denote the transpose, Hermitian, and inverse of matrix X, respectively. ∥·∥ represents the Euclidean norm of vectors and matrices, and 〈a,b〉 denotes the inner product of two vectors.

## 2. System Model

**Figure 1:**
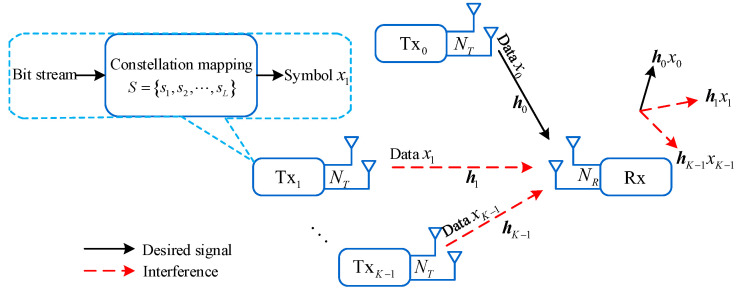
System model.

We consider a multi-user uplink communication system consisting of *K* Txs and one common Rx, as shown in Figure 1. This figure illustrates a general multi-user uplink communication model. In practice, the Rx can function as either a base station (BS) or an access point (AP), and the Txs are mobile stations. In the system model, each Tx and Rx are equipped with $N_{T}\geq 1$ and $N_{R}>1$ antennas, respectively. Assuming $N_{T}>1$, a Tx can employ either beamforming (BF) or spatial multiplexing (SM) to send one or multiple directional beams to the Rx. For simplicity, we assume that all Txs transmit to the Rx simultaneously, and their signals can arrive at the Rx synchronously. The transmit power of Tx is denoted as $P_{T}$. We use $x_{k}$ (where k∈{0,1,⋯,K−1}) to represent the data sent from ${\mathrm{Tx}}_{k}$ to Rx. $E({∥x_{k}∥}^{2})=P_{T}$ holds where E(·) denotes statistical expectation. We denote the composite channel matrix of the *K* Txs as $H=\left[h_{0}\;h_{1}\;\cdots \;h_{K-1}\right]\in C^{N_{R}\times N_{T}}$, where $h_{k}$ (k∈{0,⋯,K−1}) represents the channel matrix from ${\mathrm{Tx}}_{k}$ to Rx. We employ a spatially uncorrelated Rayleigh flat fading channel model (in this commonly used channel model, “spatially uncorrelated” is based on the assumption that the inter-antenna spacing is sufficiently large to prevent correlations among the channel fading experienced by various transmit–receive antenna pairs. Furthermore, “Rayleigh flat fading” indicates that the channel varies relatively slowly compared to the processing time of the proposed scheme. Typically, we assume that the relative speed between the Tx and the Rx is not high), where the channel elements are modeled as independent and identically distributed complex Gaussian random variables with zero mean and unit variance. We assume that each Tx, say Tx_k_, can accurately estimate $h_{k}$ and report it to the Rx via a low-rate, error-free control link. The delivery delay of CSI and signaling is negligible compared to the time scale on which the channel state varies [16,17].

As Figure 1 shows, at each Tx, the bit stream is first divided into multiple sets, and each set is converted into a data symbol through constellation mapping. This symbol is then fed to the transmit antenna for transmission. We denote the modulation symbol set used by ${\mathrm{Tx}}_{k}$ as $S=\{s_{1},\cdots ,s_{L}\}$ where card(S)=L and card(·) denotes the number of elements in the set. Here, *L* represents the modulation order, and all Txs adopt the same modulation scheme. Since we focus on the design of Rx-side processing, we set $N_{T}=1$ for simplicity. However, it is important to note that the proposed scheme can be extended to the case where $N_{T}>1$. In such instances, the Tx would need to preprocess the data before transmission.

## 3. Design of VSP-IMUD

In this section, we present the design of VSP-IMUD and describe the receiver structure employing VSP-IMUD. By exploiting interactions among wireless signals, the detection of multiple users’ signals can be achieved with fewer receiving antennas.

### 3.1. Integrated Multi-User Detection

As Figure 1 plots, when multiple Txs simultaneously send signals to the common Rx, the mixed signals received by the Rx can be expressed as

(1) $$y=\sum_{k=0}^{K-1}h_{k}x_{k}+n=r+n$$

where n denotes the Additive White Gaussian Noise (AWGN) vector whose elements have zero mean and variance $\sigma_{n}^{2}$. For clarity, we denote the mixed signal $\sum_{k=0}^{K-1}h_{k}x_{k}$ as r.

Under IMUD, the received mixed signal r is treated as a structured equivalent signal. Based on the characteristics of such an equivalent signal, the data carried in multiple signal components can be decoded simultaneously, thereby realizing MUD. Given *K* and *L*, there will be $L^{K}$ forms of r. Assuming that all Txs adopt *L*-order modulation, we can represent the modulation symbol set as $S=\left\{s_{1},\cdots ,s_{L}\right\}$, with each Tx_k_’s desired data $x_{k}\in S$. Based on the composite channel matrix H and all possible combinations of data from the *K* Txs, the Rx can derive $L^{K}$ possible forms of r, as presented in Equation (2).

(2) $$\left[r^{\left[1\right]}\;r^{\left[2\right]}\;\cdots \;r^{\left[L^{K}\right]}\right]=\left[h_{0}\;h_{1}\;\cdots \;h_{K-1}\right]\left[\begin{array}{cccc} x_{0}^{\left[1\right]} & x_{0}^{\left[2\right]} & \cdots & x_{0}^{\left[L^{K}\right]}\\ x_{1}^{\left[1\right]} & x_{1}^{\left[2\right]} & \cdots & x_{1}^{\left[L^{K}\right]}\\ \vdots & \vdots & \ddots & \vdots \\ x_{K-1}^{\left[1\right]} & x_{K-1}^{\left[2\right]} & \cdots & x_{K-1}^{\left[L^{K}\right]} \end{array}\right].$$

As Equation (2) shows, there are $L^{K}$ possible combinations of the symbol set $\left\{x_{0},x_{1},\cdots ,x_{K-1}\right\}$. Superscripts $\left[1\right],\left[2\right],\cdots ,\left[L^{K}\right]$ are used to index these symbol combinations. We denote the data combination indexed by [m] as $x^{\left[m\right]}={\left[\begin{array}{cccc} x_{0}^{\left[m\right]} & x_{1}^{\left[m\right]} & \cdots & x_{K-1}^{\left[m\right]} \end{array}\right]}^{T}$, where $m\in \{1,2,\cdots ,L^{K}\}$ and $x_{k}^{\left[m\right]}$ (k∈{0,1,⋯,K−1}) represents the data symbol sent from ${\mathrm{Tx}}_{k}$. Consequently, the *m*-th form of the received mixed signal r can be expressed as $r^{\left[m\right]}=Hx^{\left[m\right]}$. If $x^{\left[m\right]}$ is uniquely related to $r^{\left[m\right]}$, we can obtain $x^{\left[m\right]}$ by detecting the structured $r^{\left[m\right]}$ in an integrated manner.

We define the spatial feature of $r^{\left[m\right]}$ as

(3) $$g^{\left[m\right]}=\frac{r^{\left[m\right]}}{∥r^{\left[m\right]}∥}.$$

According to Equations (2) and (3), given H and $x^{\left[m\right]}$, the structure and spatial features of $r^{\left[m\right]}$ are determined. Based on Equation (3), we can employ a matched filter (MF) $g^{\left[m\right]}$ to process $r^{\left[m\right]}$. Since $x^{\left[m\right]}$ is the target that the Rx aims to detect, we need $L^{K}$ filter chains to attempt to decode the data vector $x^{\left[m\right]}$ from $r^{\left[m\right]}$. For each filter chain, we calculate the modulus of its output and select the one with the largest modulus as the correct chain. Then, we can determine the receiving filter as $g^{\left[M\right]}$ where *M* is computed in terms of $M=\underset{m\in \{1,\cdots ,L^{K}\}}{\arg\;\max}\;∥{\left[g^{\left[m\right]}\right]}^{H}r^{\left[m\right]}∥$. In this way, $x^{\left[m\right]}$ is decoded integrally. In practical applications, as *K* and/or *L* increase, the structure of SVP-IMUD becomes more complicated. To mitigate this complexity, we can divide the *K* Txs into multiple smaller groups, effectively reducing *K* before applying VSP-IMUD. Furthermore, we can increase $N_{R}$ to enhance the hardware processing capabilities at the Rx, achieving a trade-off between the number of matched filter chains and the number of receiving antennas. Additionally, it is important to note that the matched filter chains are implemented in the digital domain, eliminating the need for radio frequency chains, which makes them more cost-effective compared to receiving antennas.

**Figure 2:**
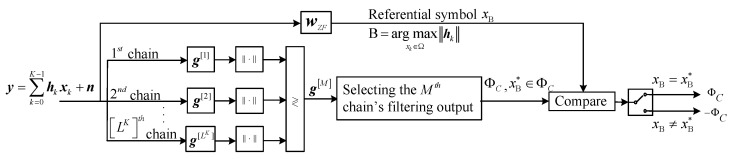
Illustration of VSP-IMUD under *K* Txs and L=2.

We denote the decoded data vector using $g^{\left[M\right]}$ as $x^{\left[M\right]}$. It is important to note that two possible forms of $x^{\left[M\right]}$, namely $x^{\left[M\right]}$ and $-x^{\left[M\right]}$, may arise due to the symmetric nature of the constellation points with respect to the origin. However, only one of $x^{\left[M\right]}$ and $-x^{\left[M\right]}$ corresponds to the correct data vector sent from the *K* Txs. To determine the exact data vector, we use ZF to recover the data from the Tx with the largest channel gain relative to the RX, and adopt this as referential data for determining the exact data vector. In Figure 2, we illustrate the realization of VSP-IMUD using binary phase shift keying (BPSK) as an example.

As the figure shows, with *K* Txs and L=2, two data vectors $x^{\left[M\right]}$ and $-x^{\left[M\right]}$ correspond to the same MF vector $g^{\left[M\right]}$. We denote these two symbol sets as $\Phi_{C}$ and $-\Phi_{C}$, respectively. Since the elements in $\Phi_{C}$ and $-\Phi_{C}$ are opposite, we use ZF to decode the data sent from the Tx with the largest channel gain to obtain the correct symbol set. We denote this referential symbol as $x_{B}$ (where B∈{0,1,⋯,K−1}). We then compare the referential symbol $x_{B}$ with the symbol under the same index in sets $\Phi_{C}$ and $-\Phi_{C}$. If there is an $x_{B}^{*}$ in $\Phi_{C}$ that matches $x_{B}$, we can determine that $\Phi_{C}$ is the correct symbol set. Otherwise, $-\Phi_{C}$ becomes the final decoding output.

In practice, due to the randomness of the Tx channels, the Euclidean distance between two rs may be very small, i.e., $∥r^{\left[i\right]}-r^{\left[j\right]}∥\leq \varepsilon$ (for $i,j\in \{1,2,\cdots ,L^{K}\}$ and i≠j), where ε is a small positive real number. This situation can lead to incorrect outputs from the Rx structure shown in Figure 2, resulting in a high bit-error rate (BER). We refer to this condition as channel ambiguity, and we will design an ambiguity detection and elimination method in the following subsection.

### 3.2. Channel Ambiguity Detection and Elimination

In the previous subsection, we assumed the structure of the received mixed signal to be unique and employed an MF to recover the data/symbol set sent from multiple TXs. In practice, however, the structure of the mixed signal may arise from various data and channel combinations, resulting in similarities and ambiguities among the characteristics of the equivalent signals. In such cases, the MF cannot achieve accurate data recovery [13].

Therefore, in this subsection, we will design a *channel ambiguity detection (CAD)* module to identify the channels that contribute to the similarity of the mixed signal’s structure, and then decode the signals transmitted via these ambiguous channels successively using SIC. Once the channel ambiguity is completely mitigated, the structure of the remaining mixed signal becomes unique, allowing for exclusive recovery of the dataset from multiple Txs using the IMUD method presented in Section III-A. Below, we will detail the detection and elimination of channel ambiguity.

We denote the set of symbols to be detected as Ω, and initialize it to $\left\{x_{0},x_{1},\cdots ,x_{K-1}\right\}$, where $x_{k}$ (for k∈{0,1,⋯,K−1}) is the data symbol sent from ${\mathrm{Tx}}_{k}$. According to Equation (2), the mixed signal perceived by the Rx is $y=\sum_{k=0}^{K-1}h_{k}x_{k}+n$. The Rx can then obtain all possible combinations of the aggregated channel matrix $H=[h_{0}\;h_{1}\;\cdots \;h_{K-1}]$ and the symbol set $\left\{x_{0},x_{1},\cdots ,x_{K-1}\right\}$. Note that although Rx does not know the desired data sent by the Txs at this time, it can still derive $\left[r^{\left[1\right]}\;r^{\left[2\right]}\;\cdots \;r^{\left[L^{K}\right]}\right]$ based on the known modulation scheme, where each element indicates a possible structure or characteristic of the received mixed signal. Next, we use the Euclidean distance between $r^{\left[i\right]}$ and $r^{\left[j\right]}$ as the ambiguity coefficient $\alpha_{\left[i,j\right]}$ of $r^{\left[i\right]}$ and $r^{\left[j\right]}$, defined in Equation (4):

(4) $$\alpha_{\left[i,j\right]}=∥r^{\left[i\right]}-r^{\left[j\right]}∥=∥\sum_{k=0}^{K-1}h_{k}\left(x_{k}^{\left[i\right]}-x_{k}^{\left[j\right]}\right)∥$$

where $i,j\in \left\{1,\cdots ,L^{K}\right\}$ and i≠j.

**Figure 4:**
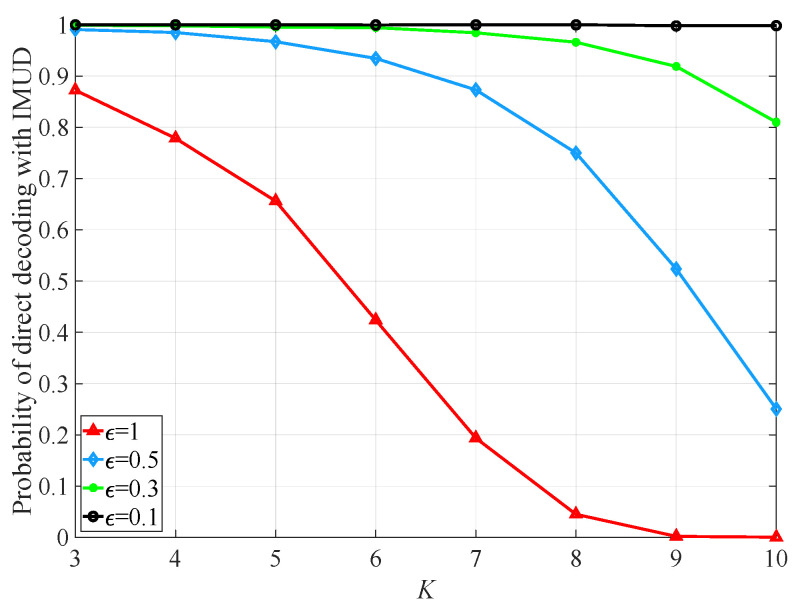
Variation of the probability of direct applying IMUD with *K* under $N_{T}=1$, $N_{R}=2$, and various εs.

We employ a real value ε>0 as the ambiguity threshold. If $\alpha_{\left[i,j\right]}\leq \varepsilon$, the structures of $r^{\left[i\right]}$ and $r^{\left[j\right]}$ are similar (ambiguous), indicating detection of ambiguity between $r^{\left[i\right]}$ and $r^{\left[j\right]}$. It should be noted that the value of ε affects the processing complexity and BER performance of the Rx. When ε is large, more pairs $\left(r^{\left[i\right]},r^{\left[j\right]}\right)$ will be classified as similar, leading to a greater number of Tx signals that need to be decoded to eliminate ambiguity, which increases the processing burden. Conversely, if ε is small, the similarities in features of the mixed signals arising from various datasets may go undetected. In this situation, while IMUD may still be applicable, it could yield an incorrect symbol set, resulting in poor BER performance. It is important to note that ε serves to balance decoding complexity and accuracy. Moreover, the degree of ambiguity depends on the *K* channel matrices associated with the *K* Txs, the *K* data symbols, and their modulation order *L*. Among these factors, the *K* channel matrices and data symbols are random and independent, making a quantitative analysis of the relationship among ε, *K* and *L* difficult. For this reason, we utilize simulations to investigate the impact of different values of ε on the probability of direct decoding with IMUD, as illustrated in Figure 4. Consequently, we can empirically determine the value of ε to achieve an optimal balance between signal processing complexity and system performance.

In order to detect the ambiguity among various pairs $\left(r^{\left[i\right]},r^{\left[j\right]}\right)$, we define an $L^{K}\times L^{K}$ ambiguity matrix F as shown in Equation (5). As depicted, the element in the *i*-th row and *j*-th column of F is either 1 or 0, indicating whether the spatial features of $r^{\left[i\right]}$ and $r^{\left[j\right]}$ are ambiguous. Specifically, if the Euclidean distance between $r^{\left[i\right]}$ and $r^{\left[j\right]}$ is less than ε, then F’s element F(i,j) is set to 1; otherwise, it is set to 0. It should be noted that under i=j, we set F(i,j)=0.

(5) $$F=\left[\begin{array}{cccccc} 0 & 1 & 0 & 0 & \cdots & 0\\ 1 & 0 & 0 & 0 & \cdots & 1\\ 0 & 0 & 0 & 0 & \cdots & 0\\ 0 & 0 & 0 & 0 & \cdots & 1\\ \vdots & \vdots & \vdots & \vdots & \ddots & \vdots \\ 0 & 1 & 0 & 1 & \cdots & 0 \end{array}\right]$$

Next, we calculate the sum of the elements in each row of F, and record the result of the *i*-th row as $\xi_{F(i)}=\sum_{j=1}^{L^{K}}F(i,j)$. The value $\xi_{F(i)}$ reflects the number of mixed signals that are similar to $r^{\left[i\right]}$. The larger $\xi_{F(i)}$ is, the more ambiguous the structure of $r^{\left[i\right]}$ is in relation to other mixed signals. In this case, if one performs IMUD on the received signal, s/he will have to choose among multiple possible symbol sets, resulting in a high BER due to this ambiguity.

The ambiguity arises from the fact that the same aggregated channel matrix $H=\left[h_{0}\;h_{1}\;\cdots \;h_{K-1}\right]$ and various symbol sets $\left\{x_{0}^{\left[m\right]},x_{1}^{\left[m\right]},\cdots ,x_{K-1}^{\left[m\right]}\right\}$ where $m\in \{1,\cdots ,L^{K}\}$ can produce identical structures for the mixed signals. To avoid ambiguity, we identify the channels in H that contribute to the ambiguity of the signal structure and subsequently remove them from H. In what follows, we will establish the criterion for determining the contribution of channel $h_{k}$ (k∈{0,1,⋯,K−1}) to the similarity between different mixed signals. We use $c_{k}$ to denote the ambiguity coefficient of $h_{k}$ and initialize $c_{k}$ to 0, indicating that $h_{k}$ does not contribute to ambiguity.

We assume that the $i^{*}$-th row of F that outputs the largest $\xi_{F(i^{*})}$ has been determined. If $F(i^{*},j)=1$, the structures of $r^{\left[i^{*}\right]}$ and $r^{\left[j\right]}$ are similar. Then, according to Equation (4), we have $\alpha_{\left[i^{*},j\right]}=∥r^{\left[i^{*}\right]}-r^{\left[j\right]}∥=∥\sum_{k=0}^{K-1}h_{k}\left(x_{k}^{\left[i^{*}\right]}-x_{k}^{\left[j\right]}\right)∥\leq \varepsilon$. Under $F(i^{*},j)=1$, if $x_{k}^{\left[i^{*}\right]}=x_{k}^{\left[j\right]}$ ($i^{*}\neq j$), we can have $h_{k}\left(x_{k}^{\left[i^{*}\right]}-x_{k}^{\left[j\right]}\right)=0$. In this case, we cannot determine the contribution of $h_{k}$ to the ambiguity of the mixed signal structures; thus, the value of $c_{k}$ remains unchanged. Conversely, if $x_{k}^{\left[i^{*}\right]}\neq x_{k}^{\left[j\right]}$ ($i^{*}\neq j$), $h_{k}\left(x_{k}^{\left[i^{*}\right]}-x_{k}^{\left[j\right]}\right)\neq 0$ holds, indicating that $h_{k}$ contributes to the structure ambiguity of the received signals. Therefore, we increment $c_{k}$ as $c_{k}\leftarrow c_{k}+1$. If $F(i^{*},j)=0$, $h_{k}$ does not contribute to the ambiguity of the received signals’ structures, and the value of $c_{k}$ remains unchanged.

After checking each $F(i^{*},j)=1$ in the $i^{*}$-th row, we can obtain the ambiguity coefficient $c_{k}$ for each $h_{k}$. Similarly, we can examine all rows of F to determine the ambiguity coefficients for the channels of all *K* Txs. We then sort $c_{k}$ ($k\in \left\{0,1,\cdots ,K-1\right\}$) in descending order. This process establishes the decoding order for the signals that generate ambiguity. Specifically, the signal transmitted via $h_{k}$ with the largest $c_{k}$, denoted as $c_{max}$, should be detected first.

Since Rx has $N_{R}$ receiving antennas, it can utilize ZF reception to concurrently decode up to $N_{R}-1$ signal components associated with the channels with the largest ambiguity coefficients in the spatial domain. For the remaining $K-N_{R}+1$ signals, they are treated as equivalent interference [11]. The Rx then employs SIC to eliminate the detected ambiguous signal components from the original mixed signal and updates the symbol set Ω by removing the decoded symbols.

Next, the Rx recalculates the ambiguity matrix and the channel ambiguity coefficients $c_{k}$ for the remaining signals and decodes any signal components that still exhibit structural ambiguity. This processing is repeated iteratively until $c_{max}=0$, at which point the IMUD method introduced in the previous subsection can be applied to the remaining mixed signals, as there will be no similarities among these components. In this way, the desired data carried in the remaining mixed signal can be uniquely determined using the spatial features of the mixed signal and the decoded referential data symbol.

It is important to note that if $N_{R}$ exceeds the number of signals to be detected, we can directly apply ZF to detect the signals. In other words, the proposed VSP-IMUD is primarily focused on scenarios where the number of signals to be detected is greater than $N_{R}$, where ZF is infeasible due to the RX’s limited number of antennas.

### 3.3. VSP-IMUD-Based Rx Structure

**Figure 3:**
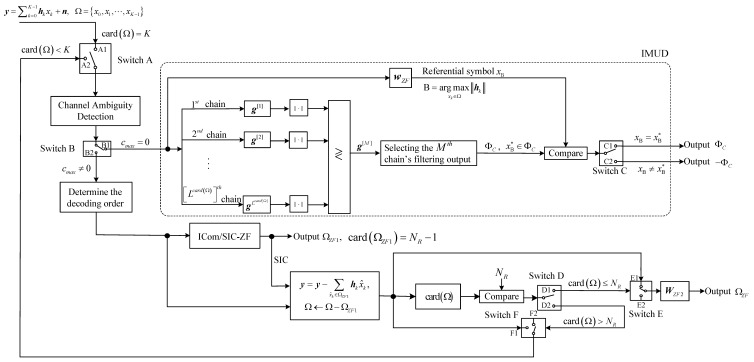
VSP-IMUD-based Rx structure.

Based on the designs of IMUD and CAD in the previous two subsections, we now present the VSP-IMUD-based Rx structure. As Figure 3 shows, there are three processing steps in the Rx structure.

***Step 1***: At Switch A, we check the relationship between the number of symbols to be decoded, denoted as card(Ω), and the total number of Txs, denoted as *K*. If card(Ω)=K, we switch to A1, and the received mixed signal is fed into the CAD module for ambiguity detection (i.e., proceed to Step 2). If card(Ω)<K, we switch A to A2 and continue with ambiguity detection for the remaining mixed signal (i.e., proceed to Step 2).

***Step 2***: For the mixed signal that has passed through the CAD module, we check $c_{max}$ at switch B. If $c_{max}=0$, we switch B to B1, and the mixed signal is fed into the IMUD module. As Figure 3 shows, there are $M=L^{card\left(\Omega \right)}$ filter chains in this structure. Each chain uses a filter vector that matches the spatial feature of a possible equivalent/structured mixed signal.

We then calculate the modulus of the output from each filter chain and determine the index of the filter chain yielding the largest modulus. Based on this index information, we can obtain two potential desired symbol sets, namely $\Phi_{C}$ and $-\Phi_{C}$. To finalize the symbol set, the Rx employs a ZF filter vector $w_{\mathrm{ZF}}$ to recover the signal from the Tx with the largest channel gain relative to the Rx, and adopts the decoded data $x_{B}$ as the referential symbol. If there is an $x_{B}^{*}$ in $\Phi_{C}$ such that $x_{B}=x_{B}^{*}$, we switch C to C1 and output $\Phi_{C}$ as the final decoded symbol set. Conversely, if $x_{B}\neq x_{B}^{*}$, we switch C to C2 and output $-\Phi_{C}$. In this way, signals from all TXs are detected.

At switch B, if $c_{max}\neq 0$, it indicates that the ambiguity of the mixed signal has not been fully resolved. Therefore, we switch B to B2 to allow the Rx to sort the channels by their ambiguity values in descending order and to detect the signals propagating through these channels. If the number of signals with $c_{k}\neq 0$ exceeds $N_{R}$, the Rx employs ICom/SIC-ZF to detect the signals from the Txs corresponding to the first $N_{R}-1$ channels with the highest $c_{k}$ values. This results in obtaining $N_{R}-1$ data symbols, which we denote as $\Omega_{ZF1}$. We can then reconstruct the detected signals and utilize SIC to subtract them from the mixed signal. After this, we can update the symbol set Ω as $\Omega \leftarrow \Omega -\Omega_{ZF1}$. Finally, we compare card(Ω) with $N_{R}$ and proceed to Step 3.

***Step 3***: At switch D, if $\mathrm{card}\left(\Omega \right)\leq N_{R}$, we switch D to D1 and concurrently switch E to E1. The Rx can then apply the ZF matrix $W_{ZF2}$ to directly detect the remaining mixed signals and obtain the detected symbol set $\Omega_{ZF2}$. This completes the MUD process. If $\mathrm{card}\left(\Omega \right)>N_{R}$, it indicates that the Rx does not have enough antennas to decode card(Ω) data. Moreover, it remains uncertain whether the ambiguity in the symbol set Ω has been mitigated. Consequently, we switch D to D2, switch F to F1, and return to Step 1, sending the mixed signal back to switch A. At switch A, since card(Ω)<K, we switch A to A2 and perform channel ambiguity detection. Based on the output from the CAD module, we switch B to either B1 or B2 (as discussed in Step 2). These steps are repeated until all *K* signals are successfully detected.

## 4. Simulation Results

In this section, we use MATLAB simulations to demonstrate the performance of VSP-IMUD. The system consists of *K* Txs and one Rx. Since we focus on a multi-user communication scenario, we set K>1 in our simulations. Although reducing *K* to 1 can eliminate CCI among multiple Txs, it also limits the system’s ability to accommodate only one user’s data transmission at a time, thereby degrading the system’s spectral efficiency (SE). Therefore, we omit the study of the single-user scenario in this work. Each Tx is equipped with $N_{T}$ antennas and employs BPSK modulation to transmit a single data stream to the Rx with power $P_{T}$. The common Rx has $N_{R}$ antennas. If $N_{T}=1$, the Tx transmits omnidirectionally; otherwise, when $N_{T}>1$, the Tx employs singular value decomposition (SVD)-based precoding to obtain the transmitted signal. In what follows, we will evaluate both the probability of direct decoding using IMUD and BER performance under VSP-IMUD. In our simulations, we assume perfect CSI for simplicity. When imperfect CSI is considered, $h_{k}$ can be re-modeled as ${\hat{h}}_{k}=\rho h_{k}+\sqrt{1-\rho^{2}}e$ [18], where $h_{k}$ and ${\hat{h}}_{k}$ denote accurate and inaccurate channel matrices, respectively. The coefficient ρ∈(0,1] indicates the degree of CSI imperfection, with ρ=1 representing perfect CSI. The matrix e is an $N_{R}\times N_{T}$ complex diagonal Gaussian matrix with zero mean and unit variance. It is important to note that all of the investigated methods are susceptible to inaccuracies in CSI. However, since the modeling of CSI imperfections and their influence on data transmission, as well as efforts to improve the algorithms’ robustness to these imperfections, have been extensively studied [19], we have omitted this discussion in the present work.

Figure 4 plots the variation in the probability of directly applying IMUD with *K* under $N_{T}=1$, $N_{R}=2$, and various εs. As the figure shows, for a fixed *K*, the probability of direct decoding with IMUD increases as ε grows. When ε is held constant, the probability increases as *K* reduces. This trend is due to the fact that a reduction in either ε or *K* decreases the detection probability of channel ambiguity, which in turn increases the likelihood of directly applying IMUD. Conversely, if ε or *K* increases, the detection probability of channel ambiguity rises, thereby reducing the probability of direct decoding with IMUD. The probability curve plotted in Figure 4 indicates that IMUD can be directly applied when no channel ambiguity is detected. Under very small ε (e.g., 0.1), the probability of directly using IMUD remains unchanged as *K* varies, as no channel ambiguity is detected.

**Figure 5:**
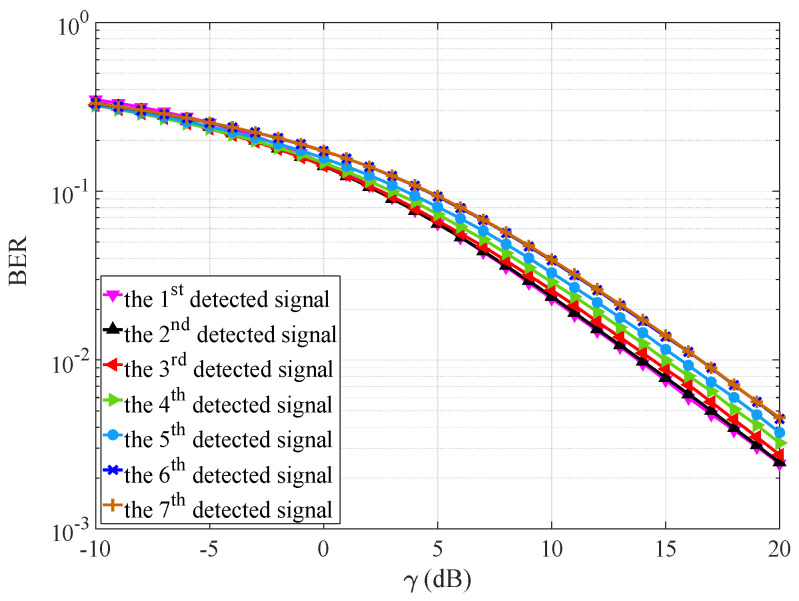
Impact of SIC on the BER of signal components (without using IMUD).

We define the transmit power normalized by noise power $\sigma_{n}^{2}$ as $\gamma =10\mathrm{lg}(P_{T}/\sigma_{n}^{2})$, and set γ∈[−10,20]dB in the simulation. Figure 5 simulates the impact of SIC on the BER of signal components under K=7, $N_{T}=1$, and $N_{R}=2$, where the Rx does not apply IMUD to decode the Txs’ data. Given $N_{R}=2$, the Rx must perform SIC five times to decode all K=7 signals until the last two signal components can be directly recovered. As illustrated in the figure, due to the error propagation issue associated with SIC, the BER for later decoded data is worse than that for previously decoded data.

**Figure 6:**
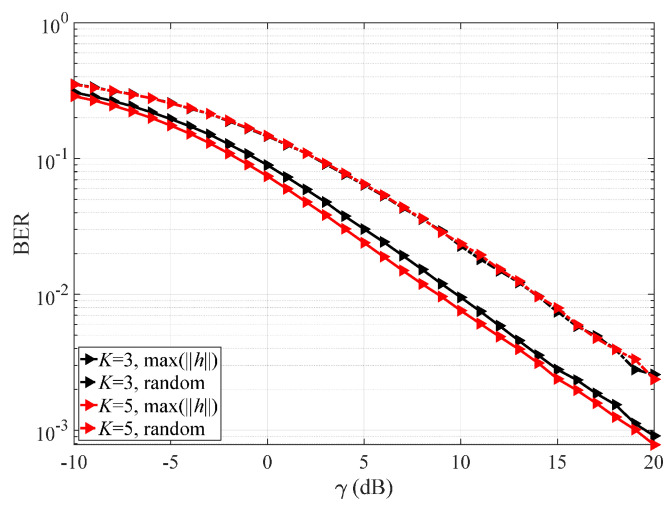
BER of VSP-IMUD under ε=0.1 using various referential symbol selection criteria.

Figure 6 displays the BER of VSP-IMUD under ε=0.1 using various referential symbol selection criteria. We use max(||h||) to indicate that the data transmitted via the channel with highest channel gain is selected as the referential symbol to determine the final symbol set. For comparison, we denote random referential symbol selection as *random*. As the figure shows, for fixed *K*, the BER using the max(||h||) criterion outperforms the random referential symbol selection. This is because random selection is more likely to produce referential symbol errors, leading to an incorrect final symbol set.

**Figure 7:**
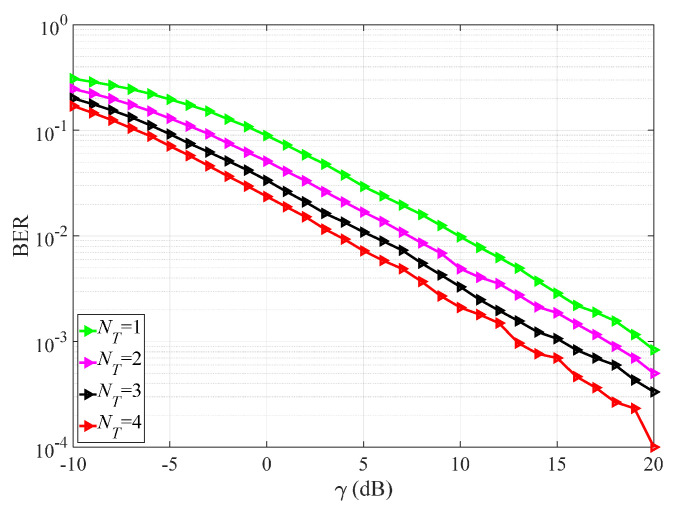
System BER of VSP-IMUD under K=3, $N_{R}=2$, ε=0.1, and various $N_{T}$s.

Figure 7 simulates the system BER of VSP-IMUD under K=3, $N_{R}=2$, ε=0.1, and various $N_{T}$s. The figure shows that the BER performance improves as $N_{T}$ increases. This improvement is due to the enhanced signal processing gain achieved by the Tx-side array when equipped with more antennas, thereby improving the overall system BER.

**Figure 8:**
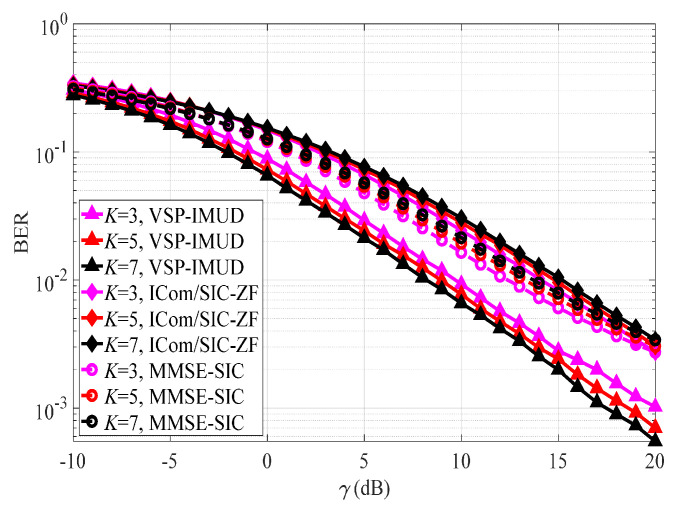
System BER of different methods under $N_{T}=1$, $N_{R}=2$, ε=0.1, and various *K*s.

Figure 8 compares the system BER of VSP-IMUD, ICom/SIC-ZF, and minimum mean square error (MMSE)-SIC under $N_{T}=1$, $N_{R}=2$, ε=0.1, and K∈{3,5,7}. We assume $K>N_{R}$ in the simulations. In this scenario, conventional ZF and MMSE become inapplicable. To make MMSE applicable when $K>N_{R}$, we treat $K-N_{R}+1$ signal components as an equivalent signal, similar to ICom/SIC-ZF. As the figure shows, under VSP-IMUD, the system BER decreases as *K* increases. This occurs because, as *K* rises, the probability of channel ambiguity occurring also slightly increases. In this situation, although the Rx needs to employ ZF to decode the signals transmitted via the ambiguous channels and subsequently use SIC to remove the detected signals from the mixed signal, the number of SIC applications remains small. That is, the increase in the probability of needing SIC is limited as *K* rises, which minimally contributes to the degradation of system BER. Furthermore, as *K* increases, the referential symbol can be selected from a larger set of candidate signals, improving the correctness of the referential symbol due to selective diversity gain. Thus, the BER performance of VSP-IMUD improves as *K* increases. In contrast, ICom/SIC-ZF and MMSE-SIC require more SIC applications as *K* grows, resulting in degraded BER performance as *K* increases. Furthermore, VSP-IMUD achieves the lowest system BER, followed by MMSE-SIC, while ICom/ZF-SIC exhibits the worst system BER. This is because the reduced reliance on SIC in VSP-IMUD enhances the decoding of multiple user signals, resulting in superior system BER compared to the other SIC-based schemes. Moreover, MMSE-SIC outperforms ICom/ZF-SIC in the medium-γ regime and converges to the performance of ICom/ZF-SIC at low- and high-γ regimes. This is because, when γ is low, noise is the dominant factor affecting the system BER; thereby, even MMSE outperforms ZF in balancing interference suppression and desired signal preservation, and both ICom/ZF-SIC and MMSE-SIC yield similar BER performance. As γ increases, CCI dominates the system BER; therefore, MMSE-SIC outperforms ICom/ZF-SIC in system BER. When γ becomes too large, ZF and MMSE exhibit similar interference suppression performance; thus, ICom/ZF-SIC and MMSE-SIC yield approximately the same system BER.

**Figure 9:**
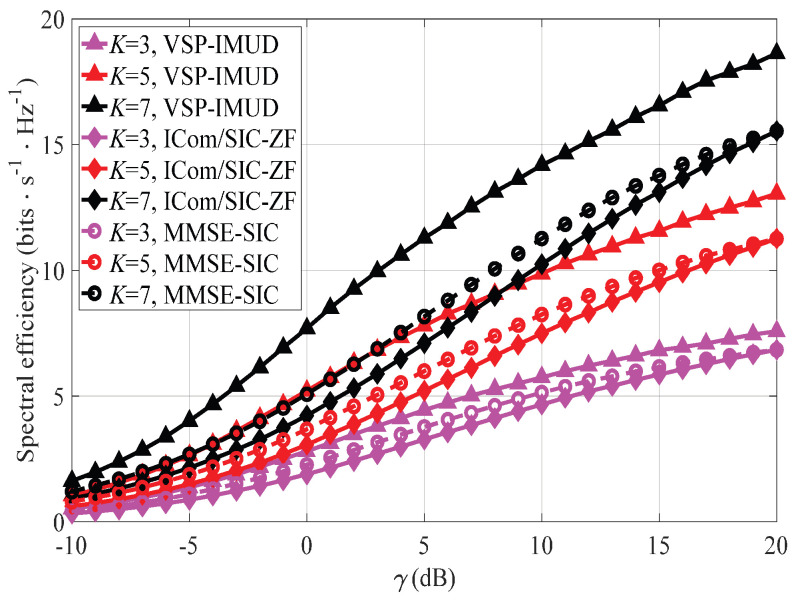
System SE of different methods under $N_{T}=1$, $N_{R}=2$, ε=0.1, and various *K*s.

Figure 9 plots the comparison of system SE of VSP-IMUD, ICom/ZF-SIC and MMSE-SIC under $N_{T}=1$, $N_{R}=2$, ε=0.1, and K∈{3,5,7}. As the figures show, VSP-IMUD achieves the highest system SE, followed by MMSE-SIC, while ICom/ZF-SIC exhibits the lowest system SE. The analysis is shown in Figure 7. Moreover, MMSE-SIC outperforms ICom/ZF-SIC in the low-γ regime and converges to the performance of ICom/ZF-SIC as γ increases. This observation is consistent with the characteristics of MMSE and ZF.

## 5. Conclusions

In this paper, we propose a VSP-IMUD-based reception scheme by exploiting the interactions among multiple wireless signals. By virtually combining multiple signal components, we can obtain an equivalent structured signal, and then employ IMUD to decode this effective signal based on its spatial characteristics. Before applying IMUD, we must detect and mitigate channel ambiguity for each signal component to ensure the uniqueness of the equivalent signal. Ultimately, we select the signal with the highest channel gain to decode a referential symbol. With this referential symbol, the Rx can output the final symbol set. Compared to other existing MUD methods, such as ICom/SIC-ZF, VSP-IMUD can effectively avoid the error propagation problem by reducing the number of SIC applications, thereby achieving improved BER performance.

## Data Availability

The original contributions presented in this study are included in the article. Further inquiries can be directed to the corresponding author.

## References

[1] Osseiran A., Boccardi F., Braun V., Kusume K., Marsch P., Maternia M., Queseth O., Schellmann M., Schotten H., Taoka H. (2014). Scenarios for 5G mobile and wireless communications: The vision of the METIS project. IEEE Commun. Mag..

[2] Liang C., He Y., Yu F., Zhao N. (2018). Enhancing video rate adaptation with mobile edge computing and caching in software-defined mobile networks. IEEE Trans. Wirel. Commun..

[3] Ercan A.Ö., Sunay M.O., Akyildiz I.F. (2018). RF energy harvesting and transfer for spectrum sharing cellular IoT communications in 5G systems. IEEE Trans. Mob. Comput..

[4] Saito Y., Kishiyama Y., Benjebbour A., Nakamura T., Li A., Higuchi K. Non-Orthogonal multiple access (NOMA) for cellular future radio access. Proceedings of the IEEE Vehicular Technology Conference (VTC).

[5] Zhu J., Xu Z., Wang F., Huang B., Zhang B. Double threshold energy detection of cooperative spectrum sensing in cognitive radio. Proceedings of the International Conference on Cognitive Radio Oriented Wireless Networks and Communication (CROWNCOM).

[6] Kalathil D.M., Jain R. (2013). Spectrum sharing through contracts for cognitive radios. IEEE Trans. Mobile Comput..

[7] Lupas R., Verdu S. (1989). Linear multiuser detectors for synchronous code-division multiple-access channels. IEEE Trans. Inf. Theory.

[8] Buzzi S., Lops M., Ricci G. A new group detection strategy for DS-CDMA systems. Proceedings of the IEEE International Symposium on Information Theory (ISIT).

[9] Moshavi S. (1996). Multi-user detection for DS-CDMA communications. IEEE Commun. Mag..

[10] Golden G.D., Foschini C.J., Valenzuela R.A., Wolniansky P.W. (1999). Detection algorithm and initial laboratory results using V-BLAST space-time communication architecture. Electron. Lett..

[11] Narasimhan R. (2005). Error propagation analysis of V-BLAST with channel-estimation errors. IEEE Trans. Commun..

[12] Li Z., Dai X., Shin K.G. Decoding interfering signals with fewer receiving antennas. Proceedings of the IEEE International Conference on Computer Communications (INFOCOM).

[13] Halperin D., Ammer J., Anderson T., Wetherall D. Interference cancellation: Better receivers for a new wireless MAC. Proceedings of the ACM Workshop on Hot Topics in Networks (HotNets).

[14] Li Z., Zhang L., Le S., Shin K.G., Liu J., Yan Z. (2025). Distributed Modulation Exploiting IRS for Secure Communications. IEEE Trans. Mob. Comput..

[15] Li Z., Zhang L., Liu C., Le S., Chen J., Shin K.G., Yan Z., Liu J. (2025). Interference Recycling: Effective Utilization of Interference for Enhancing Data Transmission. IEEE Trans. Mob. Comput..

[16] Jungnickel V., Manolakis K., Jaeckel S., Zirwas W., Panzner B., Braun V., Kottkamp M. Backhaul requirements for inter-site cooperation in heterogeneous LTE-Advanced networks. Proceedings of the IEEE International Conference on Communications Workshops (ICC).

[17] Akyildiz I.F., Gutierrez-Estevez D.M., Balakrishnan R., Chavarria-Reyes E. (2014). LTE-Advanced and the evolution to Beyond 4G (B4G) systems. Phys. Commun..

[18] Dong X., Ding Z. WLC11-3: Downlink MIMO Channel Estimation for Transmission Precoding. Proceedings of the IEEE Global Communications Conference (Globecom).

[19] Wu M., Gao Z., Huang Y., Xiao Z., Ng D.W.K., Zhang Z. (2023). Deep Learning-Based Rate-Splitting Multiple Access for Reconfigurable Intelligent Surface-Aided Tera-Hertz Massive MIMO. IEEE J. Sel. Areas Commun..

